# The interhemispheric fissure—surgical outcome of interhemispheric approaches

**DOI:** 10.1007/s10143-020-01372-6

**Published:** 2020-08-27

**Authors:** A. Kaywan Aftahy, Melanie Barz, Arthur Wagner, Friederike Liesche-Starnecker, Chiara Negwer, Bernhard Meyer, Jens Gempt

**Affiliations:** 1 Department of Neurosurgery, Klinikum rechts der Isar, School of Medicine, Technical University Munich, Ismaninger Str. 22, 81675 Munich, Germany; 2 Department of Neuropathology Klinikum rechts der Isar Institute of Pathology School of Medicine, Technical University Munich, Munich, Germany

**Keywords:** Interhemispheric approach, Meningioma, Neurosurgery, Oncology, Operative technique, Skull base

## Abstract

Exposure of the anterior skull base is challenging due to strategic structures. The interhemispheric approach (IHA) has turned out to be a feasible technique. We report our experience with IHAs in patients with extraaxial lesions (EAL). We performed a retrospective chart review at a tertiary neurosurgical center between April 2009 and March 2020. We included patients with resection of EAL through IHAs concentrating on surgical technique, complete resection rate, postoperative outcome, and complications. Seventy-four patients resected by an IHA were included: 49 (66.2%) frontal (FIA), nine (12.1%) parietooccipital (PIA), and 16 (21.6%) frontobasal IHAs (FBIAs). Median age at time of surgery was 59 years (range 16–88 years), 47 (63.5%) female and 27 (36.5%) male. Complete resection rate was 83.8% (FIA 89.8%, PIA 55.6%, FBIA 81.3%). Rate of new minor deficits was 17.6%, rate of major deficits 5.4%, total rate 23.0%. 51 (68.9%) WHO°I meningiomas, ten (13.5%) WHO°II meningiomas, two (2.7%) WHO°III meningiomas, nine (12.2%) metastases, one (1.4%) sarcoma, and one (1.4%) local adenocarcinoma were resected. Total complication rate was 27.0%. Rate of major complications requiring intervention was 9.6%. Mean follow-up was 34.2 (± 33.2) months. In patients with lesions of the interhemispheric fissure, overall morbidity and complications are comparatively high. Extensions of IHAs with potential even higher morbidity are not necessary though; we support the use of standardized IHAs. Our findings suggest regular usage of relatively feasible IHAs for a satisfying outcome. Invasive, complicated, or contralateral trajectories were not needed.

## Introduction

The frontal interhemispheric approach (FIA) exposes a variety of midline pathologies. Popularized by Ito as the anterior interhemispheric approach (IHA) to reach anterior communicating artery aneurysms [38], the technique became a standardized neurosurgical tool. The anterior dissection exposes, besides interhemispheric structures, pathologies in the suprasellar and prechiasmatic cistern including craniopharyngiomas [19, 20] and midline meningiomas, such as olfactory groove [53], planum sphenoid or tuberculum sellae meningiomas [17, 56, 74]. Access to cingular or callosal lesions through the FIA has also been described [6, 9, 21, 23, 47, 75].

As an alternative, but more as an extension, the frontobasal interhemispheric approach (FBIA) enables the view on the anterior skull base from the crista galli to the tuberculum sellae anteroposteriorly and from the midline to the sphenoid wing bilaterally. The approach has been described as safe, especially in terms of visual and pituitary stalk function [19, 27, 64]. A more subfrontal exposure is allowed.

More posteriorly, the parietooccipital interhemispheric approach (PIA) enables the resection of pathologies in the peritrigonal or periatrial region. This is challenging due to the depth of the region and due to strategically important structures [12, 37, 65].

The aim of this manuscript is to share our experience with a large series of different IHAs in patients with extra-axial oncologic pathologies at a tertiary neurosurgical university center. With the rise of new but complicated, technically challenging, poorly tested, and therefore potentially harmful approaches [9, 12–14, 73], this study aims to highlight the sufficiency of standardized IHAs and thus the reduction of perioperative morbidity.

## Materials and methods

### Study population and clinical parameters

We performed a noninterventional retrospective single-center study. Between April 2009 and March 2020, we screened the clinical documentation files and neuropathological records of patients who underwent surgery through an IHA for extra-axial tumors.

We analyzed clinical patient files for neurological symptoms, Karnofsky Performance Status Scale (KPSS), postoperative new neurological deficits, postoperative complications, reinterventions, and adverse events according to the Clavien Dindo scale (CDG). Radiological outcome parameters consisted of anatomic location as well as the extent of resection according to postoperative cranial magnetic resonance imaging (MRI) were recorded. We defined the total rate of postoperative new deficits/complications/reinterventions as the number of events divided by the number of patients. Postoperative deficits were defined as minor-to-moderate if they did not cause greater disability, whereas deficits associated with relevant loss of neurological function were defined as major. Deficits were defined as permanent if presented more than 3 months. Postoperative complications were classified in minor or moderate when no urgent intervention was needed and major in cases with subsequent intervention. A return to surgery was defined as an intervention.

We performed statistical analysis using STATA Version 13.1 (2011, StataCorp, College Station, TX). Data are shown as median and interquartile range or as mean and standard deviation; statistical significance was defined as *p* < 0.05.

### Ethics approval

The local ethics committee of the Technical University Munich, School of Medicine, approved our study (231/20 S-EB). We conducted it in accordance with the ethical standards of the 1964 Declaration of Helsinki and its later amendments [72].

## Results

### Study population

We included 74 patients who underwent surgical resection between April 2009 and March 2020. Median age of our patients at time of surgery was 59 years (range 16–88 years), with 47 (63.5%) female and 27 (36.5%) male patients. Seventeen patients (23.0%) had no preoperative symptoms; lesions were discovered incidentally during routine checkups or diagnostic workup for sinusitis or tinnitus. Fifty-seven (77.0%) patients were symptomatic, including cephalgia, nausea, diplopia, cranial nerve deficits, ataxia/imbalance, and further symptoms. The median preoperative KPSS was 90% (range 50–90), and the median postoperative KPSS 90% (range 0–100). Table 1 provides further detailed demographic and clinical information.

**Table 1:** Demographics and preoperative characteristics

Demographics % (N) or mean/median (SD/IQR)	FIA (49)	PIA (9)	FBIA (16)	Total (74)
Age	61.2 (± 14,4)	51.6 (± 16,2)	59 (± 10)	59 (± 14)
Sex	M 36.7% (18) F 63.3% (31)	M 33.3% (3) F 66.6% (6)	M 37.5% (6) F 62.5% (10)	M 36.5% (27) F 63.5 (47)
Clinical presentation				
Preoperative KPSS	90 (IQR 80–90)	90 (IQR 90–100)	90 (IQR 85–90)	90 (IQR 50–90)
Asymptomatic	20.4% (10)	22.2% (2)	31.3% (5)	23.0% (17)
Headache	18.4% (9)	33.3% (3)	25.0% (4)	21.6% (16)
Anosmia	14.3% (7)	–	37.5% (6)	17.6% (13)
Vision/visual field deficits	12.2% (6)	11.1% (1)	12.5% (2)	12.2% (9)
Hemiparesis	16.3% (8)	11.1% (1)	6.3% (1)	13.5% (10)
Paraparesis	2.0% (1)	–	–	1.4% (1)
Monoparesis		11.1% (1)	–	1.4% (1)
Hypesthesia/dysesthesia	4.0% (2)	11.1% (1)	–	4.1% (3)
Gait/stance disturbance	6.1% (3)	11.1% (1)	12.5% (2)	8.1% (6)
Seizure	26.5% (13)	–	25.0% (4)	23.0% (17)
Psychomotoric disorders	26.5% (13)	–	–	17.6% (13)
Diplopia	2.0% (1)	–	6.3% (1)	2.7% (2)
Concentration/memory/cognition impairment	2.0% (1)	–	–	1.4% (1)
Amaurosis fugax	2.0% (1)	–	–	1.4% (1)
Vertigo	10.2% (5)	22.2% (2)	25.0% (4)	14.9% (11)
Aphasia/dysarthria	2.0% (1)	–	–	1.4% (1)

*FBIA*, frontobasal interhemispheric approach; *FIA*, frontal interhemispheric approach; *KPSS*, Karnofsky Performance Status Scale; *PIA*, parietooccipital interhemispheric approach

### Tumor entities, location, and approach-related findings

Histopathological analysis revealed 51 (68.9%) cases with WHO°I meningiomas, ten (13.5%) WHO II meningiomas, two (2.7%) WHO°III meningiomas, nine (12.2%) metastases, one (1.4%) sarcoma, and one (1.4%) local adenocarcinoma. Table 2 shows all entities and performed approaches in detail. FIA was mostly performed for falcine/parafalcine lesions (69.4%) and for lesions at the olfactory groove (22.4%). PIA was also used most commonly for falcine/parafalcine lesions of the middle or last third of the superior sagittal sinus (55.5%) but also for metastases in the cingular gyrus. The FBIA was additionally used for technically demanding basal midline meningiomas of the anterior skull base as well as for frontobasal falcine tumors. Table 3 displays the tumor location in relation of the described approaches.

**Table 2:** Histopathological findings, locations and performed approaches

	FIA (49)	PIA (9)	FBIA (16)	Total (74)
Tumor entity % (*N*)				
Meningioma WHO grade I	64.7% (33)	66.7% (6)	75.0% (12)	68.9% (51)
Atypic meningioma WHO grade II	16.3% (8)	–	12.5% (2)	13.5% (10)
Anaplastic meningioma WHO grade III	4.1% (2)	–	–	2.7% (2)
Metastasis	10.2% (5)	33.3% (3)	6.3% (1)	12.2% (9)
Sarcoma	2.0% (1)	–		1.4% (1)
Adenocarcinoma	–	–	6.3% (1)	1.4% (1)
Tumor location % (*N*)				
Falcine/parafalcine	69.4% (34)	55.5% (5)	50.0% (8)	63.5% (47)
Olfactory groove	22.4% (11)	–	37.5% (6)	23.0% (17)
Planum sphenoidale	–	–	6.3% (1)	1.4% (1)
Frontobasal	–	–	6.3% (1)	1.4% (1)
Tentorial	–	11.1% (1)	–	1.4% (1)
Cingular gyrus	2.0% (1)	11.1% (1)	–	2.7% (2)
Central region	4.1% (2)	11.1% (1)	–	4.1% (3)
Thalamic	2.0% (1)	11.1% (1)	–	2.7% (2)

*FBIA*, frontobasal interhemispheric approach; *FIA*, frontal interhemispheric approach; *PIA*, parietooccipital interhemispheric approach; *WHO*, World Health Organization

**Table 3:** Extent of resection, postoperative presentation and outcome

Postoperative presentation % (N)	FIA (49)	PIA (9)	FBIA (16)	Total (74)
Complete resection (Simpson I + II + GTR)	89.8%	55.6%	81.3%	83.8%
EOR in case of other than meningioma	GTR 66.6% (4/6) STR 33.3% (2/6)	GTR 66.6% (2/3) STR 33.3 (1/3)	GTR 50% (1/2) STR 50% (1/2)	GTR 63.6% (7/11) STR 36.4% (4/11)
Simpson grades I–IV in case of meningioma	I 58.1% (25/43) II 34.9% (15/43) III 2.3% (1/43) IV 4.7% (2/43)	I 50.0% (3/6) III 16.7% (1/6) IV 33.3% (2/6)	I 35.7% (5/14) II 50.0% (7/14) III 14.3% (2/14)	I 52.4% (33/63) II 34.9% (22/63) III 6.3% (4/63) IV 6.3% (4/63)
Intraoperative complications	- Pericallosal artery injury 2.0% (1)	- -	MEP decrease 6.25% (1)	MEP decrease 1.4% (1) Pericallosal artery injury % 1.4 (1)
Postoperative complications	Minor complications	Minor complications	Minor complications	Minor complications
	Venous congestion 6.2% (3) Hydrocephalus 2.0% (1)	Venous congestion 11.11% (1) -	WHD 6.3% (1) - -	WHD 1.4% (1) Venous congestion 5.4% (4) Hydrocephalus 1.4% (1)
	Major complications	Major complications	Major complications	Major complications
	EDH 6.2% (3) SDH 2.0% (1) ICH 4.1% (2)	- - -	- - ICH 6.3% (1)	EDH 4.1% (3) SDH 1.4% (1) ICH 4.1% (3)
	During follow-up	During follow-up	During follow-up	During follow-up
	Abscess 6.2% (3)	Abscess 11.1% (1)	Abscess 18.8% (3)	Abscess 9.5% (7)
Postoperative interventions	- Revision due to hemorrhage 8.2% (4) Revision due to infection 4.1% (2) VP-Shunt 2.0% (1) Bone explant/implant 2.0% (1)	- - - - Bone explant/implant 11.1% (1)	Wound revision 6.3% (1) - - Revision due to infection 12.5% (2) - Bone explant/implant 6.3% (1)	Wound revision 2.7% (1) Revision due to hemorrhage 5.4% (4) Revision due to infection 5.4% (4) VP-Shunt 1.4% (1) Bone explant/implant 4.1% (3)
New permanent neurological deficits	Minor deficits	Minor deficits	Minor deficits	Minor deficits
	Visual deficits 2.0% (1) Hemiparesis 12.2% (6) Psychomotoric disorders 2.0% (1)	- Hemiparesis 11.1% (2) Paraparesis 11.1% (1) -	- Hemiparesis 6.3% (1) - Psychomotoric disorders 6.3% (1)	Visual deficits 1.4% (1) Hemiparesis 12.2% (9) Paraparesis 1.4% (1) Psychomotoric disorders 2.7% (2)
	Major deficits	Major deficits	Major deficits	*Major deficits*
	Neglect 2.0% (1)	-	-	Neglect 1.4% (1) Aphasia/dysarthria 1.4% (1) Hemiplegia 2.7% (2)
	Aphasia/dysarthria 2.0% (1) Hemiplegia 2.0% (1)	-	- Hemiplegia 6.3% (1)	
Postoperative KPSS	80% (0–90)	90% (70–100)	90% (80–90)	90% (0–100)
CDG	I 59.2% (29) II 18.4% (9) IIIa 6.1% (3) IIIb 12.2% (6) IV 2.0% (1) V 2.0% (1)	I 66.6% (6) II 22.2% (2) - IIIb 11.1% (1) - -	I 68.8% (11) - IIIa 12.5% (2) IIIb 18.8% (3) - -	I 62.2% (46) II 14.9% (11) IIIa 6.8% (5) IIIb 13.5% (10) IV 1.4% (1) V 1.4% (1)
Follow-up time in months	33.4 (± 34.7)	31 (± 35.7)	38.2 (± 29.3)	34.2 (± 33.2)

*CDG*, Clavien Dindo Scale; *EDH*, epidural hematoma; *FBIA*, frontobasal interhemispheric approach; *FIA*, frontal interhemispheric approach; *GTR*, gross total resection; *ICH*, intracerebral hemorrhage; *KPSS*, Karnofsky Performance Status Scale; *MEP*, motor evoked potential; *PIA*, parietooccipital interhemispheric approach; *SDH*, subdural hematoma; *STR*, subtotal resection; *VP*, ventriculoperitoneal; *WHD*, wound healing disorder

### Functional outcome and surgical complications

Simpson grade I + II resection was achieved in 87.3% (FIA 93.0%, PIA 50.0%, FBIA 85.7%) regarding meningiomas. Complete resection (Simpson grades I + II and GTR) was achieved in 83.8% of the patients (FIA 89.8%, PIA 55.6%, FBIA 81.3%) (Table 3)**.**

Postoperative rate of new permanent minor-to-moderate neurological deficits was 17.6%. Most frequently, hemiparesis was observed in nine patients (12.2%), followed by psychomotoric disorders in two patients (2.7%). New postoperative hemianopsia was observed in one case. Regarding major deficits, postoperative hemiplegia was observed in two (2.7%), followed by dysarthria and neglect in one (1.4%) patient.

One patient with falcine meningioma of the anterior third of superior sagittal sinus committed suicide postoperatively. In another case with falcine meningioma, resected through a FIA, the ipsilateral pericallosal artery was injured and subsequently sacrificed, leading to a new postoperative permanent hemiparesis.

The overall postoperative complication rate was 27.0% (FIA 26.5%, PIA 22.2%, FBIA 31.3%) The rate of postoperative major complications was 9.6% The majority of postoperative hemorrhages/hematomas (epidural hematoma 4.1%, subdural hematoma 1.4%, intracerebral hemorrhage 4.1%) with subsequent revision occurred after a FIA (FIA 12.0%, PIA 0.0%, FBIA 6.3%). Most common reinterventions were revisions due to hemorrhage or infections in four (5.4%) patients, respectively. FBIA developed more postoperative abscesses (FBIA 18.8%, PIA 11.1%, FIA 6.2%). None of the abscesses (9.5%) occurred directly postoperative but rather during the first three postoperative months. A revision due to postoperative infection was necessary in 4.1% of the patients resected by FIA and in 12.5% of FBIAs (Table 3). Total reintervention rate was 17.6% (FIA 16.3%, PIA 11.1%, FBIA 25.0%). However, no statistical significance could be detected regarding approach-related complications (*p* > 0.05).

**Fig. 1:**
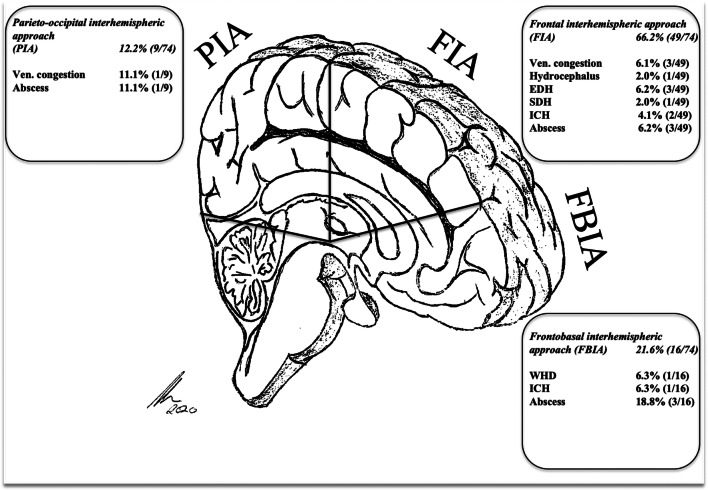
Approach-related complications % (N). Abbreviations: WHD, wound healing disorder; EDH, epidural hematoma; SDH, subdural hematoma; ICH, intracerebral hemorrhage

Proportionally, postoperative venous congestion occurred in 11.1% of the patients after a PIA, followed by 6.3% after a FIA *(p* > 0.05). None of them needed operative reintervention in the postoperative course. Venous congestion, shunt-dependent hydrocephalus, and wound healing disorders were classified as moderate or minor complications (8.1%) as no immediate return to surgery was needed. (Fig. 1).

The Clavien Dindo Scale for postoperative adverse events showed grade I in 46 (62.2%), grade II in eleven (14.9%), and grade IIIb in ten (13.5%) patients (Table 3). Mean follow-up was 34.2 (± 33.2) months.

## Discussion

Our series showed that the IHA is a technically and comparably safe operative technique. Despite new, but complicated and technically challenging approaches [9, 12–14, 73], we could achieve complete resection (Simpson grades I + II and GTR) in 83.8% of our cases (exemplary cases 1–2) with standardized IHAs.

Rate of total new permanent neurological deficits (severe: 5.4%, minor or moderate: 17.6%) was 23.0% (FIA 22.4%, PIA 33.3%, FBIA 18.8%), and total complication rate was 27.0% (FIA 26.5%, PIA 22.2%, FBIA 31.3%), satisfactory results compared with the findings of other previous experiences [5, 12, 13, 18, 27, 45, 56, 64, 67]. However, approach-related obstacles and complications must be taken into consideration when choosing the right approach, anatomic approach–related knowledge is necessary to avoid unnecessary increasing complication rates.

Our findings showed 26.5% and 22.2% for FIA and PIA, respectively, which are satisfactory results and are in line within the range of the findings of other authors, such as 24.1% by Liu et al., 11.5% by Mielke et al., 22.6% by Spicer et al., or even 36.5% by Aryan et al. Actually, in our series, rate of postoperative major complications requiring surgical intervention was 9,6%. Direct comparison with the other findings still remains difficult as others either focused on specific entities, infants or included trauma cases, and vascular lesions [5, 12, 25, 44–46, 48, 67, 74].

For decades, several authors have described alternative approaches to reach, e.g., suprasellar meningiomas or midline lesions of the anterior skull base as an alternative to the IHA.

Median approaches, as presented in current series, might facilitate intraoperative orientation as anatomy is visualized in a straight line. The classic bifrontal approach allows good devascularization, especially in case of frontobasal midline meningiomas [1, 2, 8, 50, 52, 56, 61, 62]. Still visualization of the anterior communicating complex and optic nerves might be laborious as they are hidden behind the tumor masses which might render this approach less suitable for tuberculum sellae meningiomas [11, 36, 40, 52]. The FIA/FBIA might optimize the control of the anterior vascular structures and optic nerves but has been criticized for possible parenchymal retraction [39, 46]. An adoption from the class bifrontal approach is the transbasal/subfrontal approach, a frontal lobe preserving approach initially described by Cushing [17, 44, 70] which enables superior devascularization with less brain retraction. Wide exposure and unilateral sparing of the contralateral frontal lobe have been described as some of the advantages [20, 24, 51, 52, 56, 66], but pitfalls of that approach like opening of frontal sinuses and the invasivity with inferior border osteotomies sometimes not being sufficient enough to avoid brain elevation and making neurovascular dissection necessary have let more authors emphasize the advantages of dissecting the interhemispheric fissure for wider exposure. In addition, the risk for CSF leakage might be increased [15, 26, 42, 52, 54–56, 60]. In contrast, other authors advocate more technically demanding transbasal approaches to reach the anterior skull base [20, 24, 66]. Therefore, the FBIA enables a broader anatomical corridor of the interhemispheric fissure that might reduce frontal lobe retraction [19, 20, 27, 56, 63, 69]. The FBIA might offer a better visualization if the boney skull base is infiltrated [19, 20, 27, 56, 63, 69]. Nakamura et al. also showed a higher total resection rate (Simpson grade 1 or 2) through a median approach in their series of 82 OGMs: 91.2% (frontolateral) vs. 93.5% (bifrontal) [52]; however, they still advocate the less invasive frontolateral approach due to experienced lower morbidity rate and no mortality.

Lateral approaches allow for satisfactory and good visualization of the anterior circulation on both sides as well as superior access to the optic nerve, chiasm, and pituitary complex [1, 11, 35, 49, 52, 74]. The classic pterional and frontolateral approach [35, 61, 74] offers wide visualization of parenchyma and skull base especially for lesions extending more laterally. As for intradural preparation, meticulous drilling and irrigation is necessary in order to avoid heat related damage to the optic structures [51, 52, 54]. Various modifications of these “working horses” in skull base surgery have been published and trended over recent years [4, 16, 32, 33, 57]. Purely endoscopic or endoscopy assisted resections via transnasal or transcranial routes have been popularized for some time offering minimal invasive visualization of the skull base region [49, 59]. This trend has not peaked into a relevant paradigm shift as extent of resection, technical feasibility, and CSF leakage rates have been described to be unfavorable compared with classic skull base approaches [7, 41, 49, 59]. Therefore, we do not see any reasonable indication to perform such techniques. Otherwise, if endoscopic approaches had led to any noteworthy advantages, such techniques would have been performed more regularly and spaciously.

However, regarding FIA, we observed postoperative hemorrhages and hematomas as noteworthy complications (12.0%). Three epidural, one subdural hematoma, and two intracerebral hemorrhages occurred. Brain retraction as a potential factor for postoperative intracerebral hemorrhages should be consequently reduced as much as possible. Furthermore, the craniotomy and the chosen trajectory should be carefully adapted to the planned approach in order to reduce retraction. Intraoperative meticulous hemostasis as well as closure and usage of tenting sutures, for example, may be taken into consideration as well, especially for the prevention of sub- and epidural hematomas [71, 76]. The FIA remains a suitable approach for large meningiomas of the anterior cranial base, even with involvement of the anterior cerebral arteries into the posterior aspect of the tumor, due to an optimized view from superior–posterior and superior–anterior.

Regarding the PIA, one should notice that crucial vascular structures such as important central bridging veins, the vein of Trolard, and the Rolandic vein are located nearby. Preservation is mandatory during resection, even more than in FIAs, as more of the central region is exposed when more posterity is chosen. Those veins still limit the dural opening of the already narrow working space [43, 58, 68]. We also experienced such difficulties. This complication may be one of the main challenges of the PIA. The importance of preserving bridging veins is not negligible. The study of preoperative MRI to detect prominent bridging or anastomotic veins is essential for sufficient planning.

Major deficits, such as neglect, aphasia, and hemiplegia, respectively, could be considered as a result of exposure of the central region or the parietal lobe, too much brain retraction but also due to damage of frontal and central bridging veins, or ligation of the superior sagittal sinus. Aryan et al. showed that incidence of transient postoperative hemiparesis in infants appeared to be higher in those who required ligation of one or two parasagittal veins (44.6% versus 18.5%) in their series of 65 IHA in infants [5] (exemplary case 3). Spicer et al. described required coagulation and lysis of bridging cortical veins from the convexity to the sagittal sinus in 41.5% in their series of 53 cases of IHAs for mass lesions in childhood, which resulted in postoperative hemiparesis in 30% [67]. Exposure of the superior sagittal sinus is correlated with a better angle of view [3], but also increases the risk of injury of the sinus and the necessity of sacrificing parasagittal/bridging veins, as seen above, so that especially in the middle and posterior segment the preservation of integrity of the superior sagittal sinus and related bridging veins is of utter importance for the postoperative outcome. In cases of postoperative venous congestion, a low-dose heparinization and further change to low-molecular-weight heparin should be taken into consideration. Especially in case of asymptomatic patients, we experienced no need of extensive postoperative heparinization. Recent retro- and prospective studies also showed higher radiographic incidence of postoperative cerebral venous sinus thrombosis than reported in retrospective studies [10, 22, 29, 30]. They also advocated conservative treatment in absence of symptoms; collateral venous drainage is discussed as one of the main mechanisms for well thrombosis tolerance.

To conclude, exposure of superior sagittal sinus, the central region, sacrificing bridging veins, especially parietally, and retraction in case of suboptimal approach and trajectory planning has to be minimized to fully enable the advantage of this comparably cortex sparing technique.

As an extension of the conventional IHA, FBIA can be chosen if the right indication is given and if pathoanatomical conditions require this more extensive approach, such as for large midline meningiomas or craniopharyngiomas [19, 20, 27, 56]. Primary described by Suzuki et al. as a variation of the bifrontal anterior interhemispheric approach for 3rd ventricle tumors and anterior communicating artery aneurysms, the FBIA has been extended and modified in the last decades to expose the anterior skull base from a more subfrontal view [63, 69]. The FBIA has all advantages of the classic anterior subfrontal approach and of the FIA by using the anatomical corridor of the interhemispheric fissure. Frontal lobe retraction can be minimized if the fissure is sufficiently divided. This kind of approach can expose the complete tumor height. Ganna et al. could show satisfactory postoperative outcomes in their series of resected tuberculum sellae meningiomas by FBIA, especially no new visual deficits were observed in their patient cohort, which reflects the outcome of our patient group [27].

Surgically, early identification of the optic apparatus is essential; in most cases, the optic nerves are found displaced posteriorly laterally. After initial tumor debulking further coagulation may be done cautiously as carotid artery often could be placed medial to the displaced optic nerves. Due to already mentioned advantages of exposure and point of view by the FBIA, retraction of the optic nerves is normally not necessary. However, attention must be paid to any arachnoid plane as it serves as a natural layer between the tumor and the neurovascular structures; mobilization and complete tumor removal are possible but may be refrained in case of greater adherence in order to maintain visual function. In some cases, the chiasmatic and lamina terminalis cistern has to be exposed more extensively. Afterwards, the anterior communicating complex will come into view and enables more intraoperative control. Unroofing the optic canal and incision of the falciform ligament may be required in case of tumor infiltration into the optic canal and in case of preoperative visual impairment. Such decompression is not mandatory in every case as drilling the optic roof, and the tuberculum sellae may cause additional CSF leak.

To sum it up, we emphasize detailed exposure and wide interhemispheric dissection prior to sometimes too early tumor resection in order to expose, view, and therefore control all necessary structures.

In our series, FBIA is associated with highest approach-related complication rate (FIA 26.5%, PIA 22.2%, FBIA 31.3%), most likely caused by the most invasive nature of this technique compared with the others possible frontal sinus opening. On the other hand, FBIA results with least rate of new deficits (FIA 22.4%, PIA 33.3%, FBIA 18.8%).

Regarding complications, cerebrospinal fluid (CSF) leaks are considered the most common ones through such frontobasal techniques, similar to transbasal approaches, especially after dural reconstruction [15, 26]. However, we experienced no postoperative CSF leak at all, satisfactory results compared with the findings of for example Obeid et al. with 20% or Raveh et al. and Kurtsoy et al. with 4.8% and 6.0%, respectively [42, 56, 60], but one should notice that they referred to more transbasal approaches than we did. The basic surgical technique of the FBIA is already described in detail by other authors before [27, 64]. In fact, we did not use any noteworthy alterations of known techniques, but we advocate regular usage of a navigation system, if applicable, as frontal sinus can be detected preciously and possibly be spared out during craniotomy. We experienced that in some cases, full bony basal exposure is not that mandatory as interhemispheric dissection and the angle of view from anterior to superior or, after new table and microscope positioning, even posterior–superior, is satisfactory. Of course, in case of greater lesions with even infiltration of the anterior skull base, full exposure is necessary. In such cases, besides cranialization of the sinus, meticulous dural reconstruction and skull base coverage must be performed, whereas we prioritize priory preparation und usage of a galea-periosteum-flap. We moved away from using fascia lata in the first place as we experienced more CSF leaks than with the flap, which may be based on insufficient vascularization. Necrosis and thus also source of superinfections may be further consequences. However, this just reflects our own institutional experience, whereas techniques of anterior skull base reconstruction have been extensive discussed and algorithms proposed [28, 31, 34].

Anyway, comparison of complication rates is difficult as other authors referred to specific entities, outcome parameters or were small sample sized [20, 27, 45, 64].

**Fig. 2:**
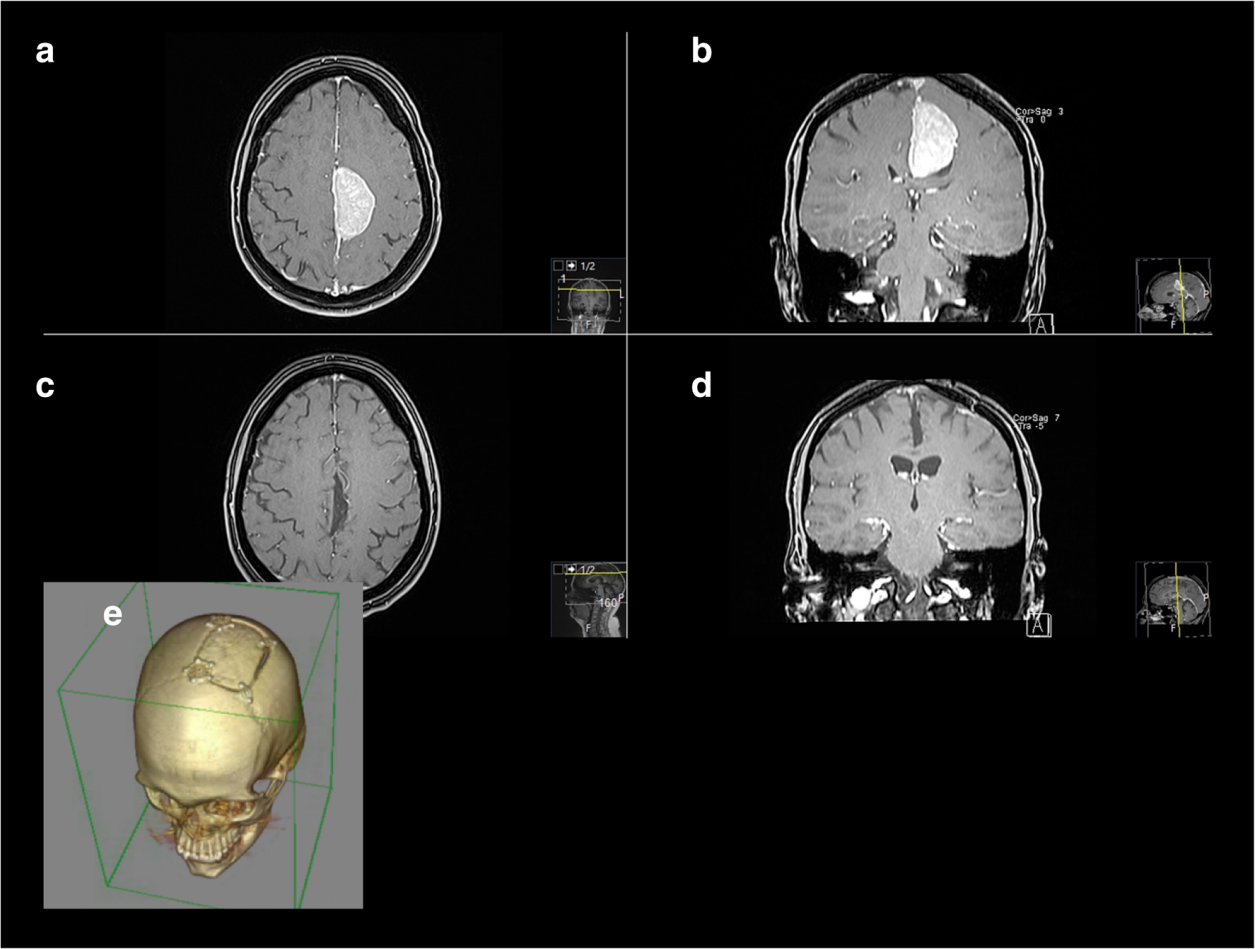
Exemplary case 1: A 35-year-old female patient presented with depression and seizures. **a**, **b** Preoperative T1-weighted gadolinium–enhanced MR imaging showing a left-sided falcine meningioma. **c**, **d** Postoperative MR control showing complete removal, Simpson I. Pathologic findings revealed atypic meningioma WHO grade II. **e** Postoperative 3D-CT reconstruction showing the bone flap for a FIA

**Fig. 3:**
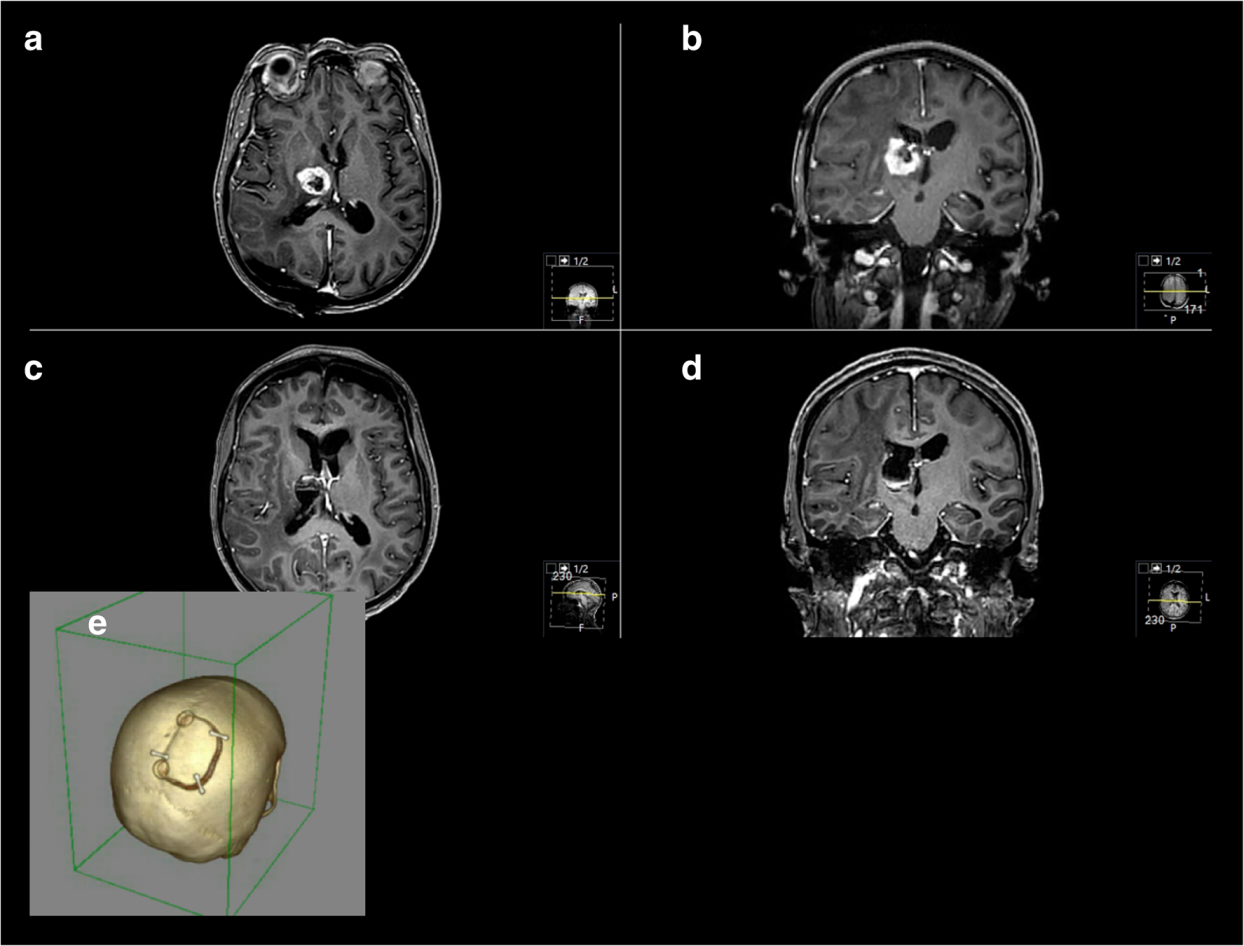
Exemplary case 2: A 52-year-old female patient presented with paresis. Known breast cancer. **a**, **b** Preoperative T1-weighted gadolinium-enhanced MR imaging showing a cystic right thalamic tumor. **c**, **d** Postoperative MR control showing successful resection. Pathologic findings confirmed the metastasis of breast cancer. **e** Postoperative 3D-CT reconstruction showing the bone flap for a PIA

**Fig. 4:**
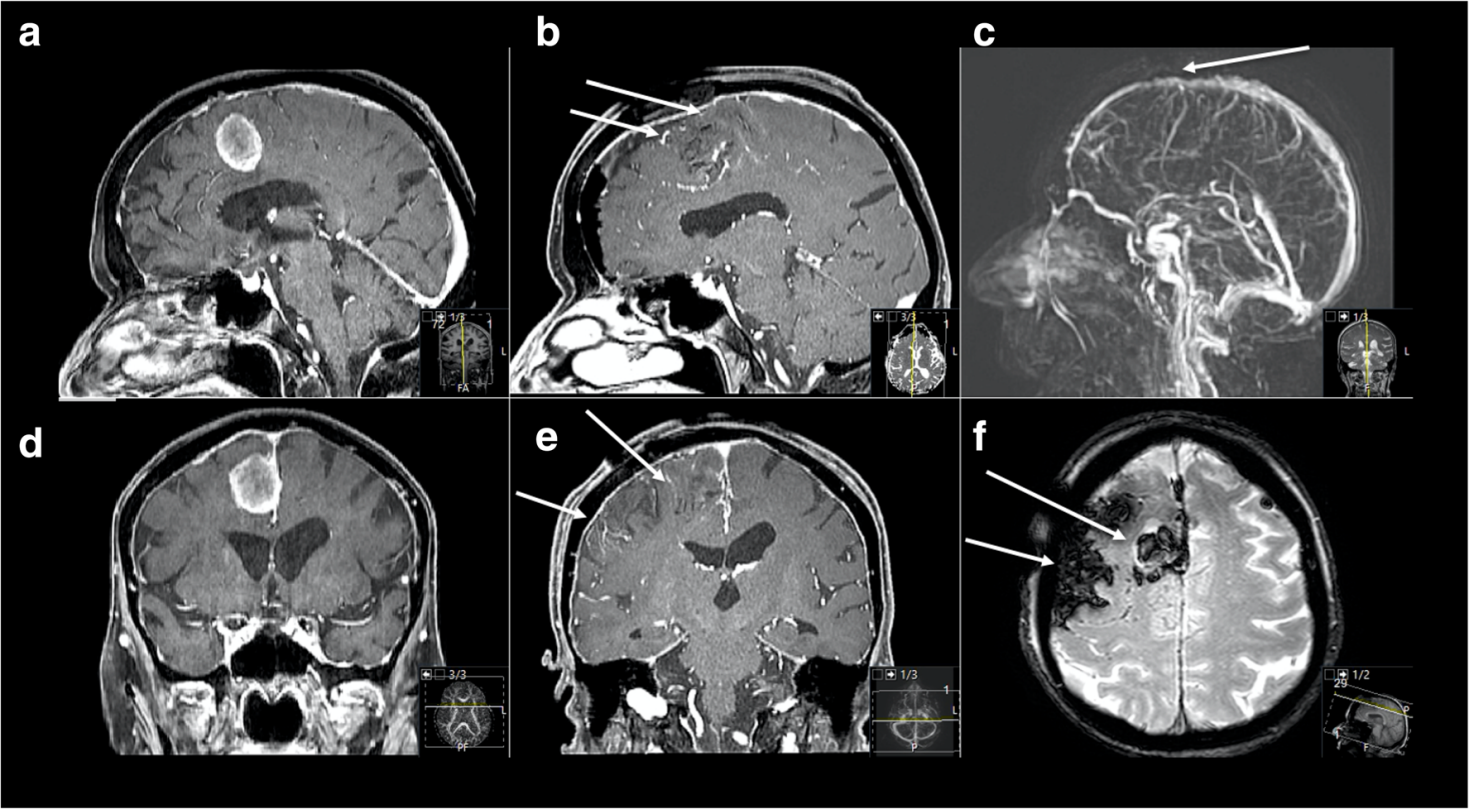
Exemplary case 3: A 75-year-old female patient presented with slight progressive headache, psychomotoric decline, vertigo, and subjective weakness of left lower limb. **a**, **b** Preoperative T1-weighted gadolinium–enhanced MR imaging showing a right-sided falcine meningioma with maximal diameter of 2.4 cm. A FIA was performed. **c**, **d** Postoperative MR control showing successful resection, but with new right frontal parenchymal hemorrhage as well as bleeding into the resection cavity. Increased vessel drawing in the sulci as indirect sign of postoperative venous congestion (arrows) can be seen. **e** Postoperative dynamic MR angiography reconstruction showing no obvious bridging or sinus vein thrombosis, but certain constriction is visible (arrow). **f** Multiecho gradient recalled echo (GRE) T2*-weighted imaging showing typical hemorrhage configurations with accompanying visible vessels along the sulci and gyral edema (arrows). Postoperatively, the patient suffered from deteriorated psychomotoric decline and new left-sided lower limb pronounced hemiparesis which did not recover completely during follow-up. However, walking and daily activities were possible in further course

Compared with the other approaches, we observed a noteworthy frequency of infection-related complications by FBIA. Incidence of infection is relatively low [15, 26, 28, 45, 60, 62]. They may occur in the case of larger tumors and longer operative times requiring surgical revision with bone flap explantation and plastic flap reconstruction, as with one of our patients (6.3%). Accidental opening of frontal sinus and consecutive contamination, especially in the case of sinusitis, must be taken into consideration (Figs. 1, 2, 3 and 4). Careful cranialization and sinus obliteration with removal of the mucosa, if needed, should be performed to minimize the postoperative infection rate. In the case of prominent sinus, sinusitis, or other active nasosinal infections, another approach may be considered.

### Study limitations

As it is a retrospective case series, it is not possible to draw causalities with respect to clinical outcome. Nevertheless, we implemented detailed clinical examination, including scores on functional performance, and a standardized follow-up protocol based on a certified neurooncological board into our clinical workflow. Nevertheless, the current study has some noteworthy limitations. Besides of its retrospective nature, the analyzed patient collective suffers from certain aspects of heterogeneity. First, the PIA is limited to 9 on a total of 74 which could lead to variability in the results. We decided to include the PIA into analysis as all aspects of interhemispheric approaches should be reflected and as basic surgical techniques do not differ exceptionally between FIA and PIA.

Secondly, variability of pathologies included could lead to further heterogeneity as majority of cases were meningiomas. With aiming at the approach related complications, we decided to focus on extrinsic lesions, as manipulation techniques seemed to be more similar, so complications could be reduced on the approaches as good as possible.

## Conclusions

In patients with lesions of the interhemispheric fissure, overall morbidity and complications are comparatively high. Modifications or extensions of IHAS with a potential even higher morbidity are not necessary; we support the use of standardized IHAs for a variety of entities. Our findings suggest regular usage of relatively feasible IHAs for a satisfying outcome and maximal extent of resection. Invasive, complicated, or contralateral trajectories were not needed.
